# Transcriptome analysis of differentiating trypanosomes reveals the existence of multiple post-transcriptional regulons

**DOI:** 10.1186/1471-2164-10-495

**Published:** 2009-10-26

**Authors:** Rafael Queiroz, Corinna Benz, Kurt Fellenberg, Jörg D Hoheisel, Christine Clayton

**Affiliations:** 1 Zentrum für Molekulare Biologie der Universität Heidelberg, ZMBH-DKFZ Alliance, Im Neuenheimer Feld 282, 69120 Heidelberg, Germany; 2 Deutsches Krebsforschungszentrum, In Neuenheimer Feld 280, 69120 Heidelberg, Germany; 3 Division of Infection & Immunity and Wellcome Trust Centre for Molecular Parasitology, Glasgow Biomedical Research Centre, 120 University Place, Glasgow, G12 8TA, UK; 4 Centre for Integrated Protein Sciences Munich (CIPSM), Lehrstuhl für Bioanalytik, Technische Universität München, an der Saatzucht 5, 85354 Freising, Germany

## Abstract

**Background:**

Trypanosome gene expression is regulated almost exclusively at the post-transcriptional level, with mRNA degradation playing a decisive role. When trypanosomes are transferred from the blood of a mammal to the midgut of a Tsetse fly, they transform to procyclic forms: gene expression is reprogrammed, changing the cell surface and switching the mode of energy metabolism. Within the blood, trypanosomes can pre-adapt for Tsetse transmission, becoming growth-arrested stumpy forms. We describe here the transitions in gene expression that occur during differentiation of *in-vitro *cultured bloodstream forms to procyclic forms.

**Results:**

Some mRNAs showed changes within 30 min of *cis-*aconitate addition, whereas others responded 12-24 hours later. For the first 12 h after addition of *cis*-aconitate, cells accumulated at the G1 phase of the cell cycle, and showed decreases in mRNAs required for proliferation, mimicking the changes seen in stumpy forms: many mRNAs needed for ribosomal and flagellar biogenesis showed striking co-regulation. Other mRNAs encoding components of signal transduction pathways and potential regulators were specifically induced only during differentiation. Messenger RNAs encoding proteins required for individual metabolic pathways were often co-regulated.

**Conclusion:**

Trypanosome genes form post-transcriptional regulons in which mRNAs with functions in particular pathways, or encoding components of protein complexes, show almost identical patterns of regulation.

## Background

African trypanosomes grow in various mammalian hosts and in Tsetse flies, and are extracellular throughout their life cycle. Within the mammal, the cells grow as long slender trypomastigotes in the blood and tissue fluids, depending on glucose and substrate-level phosphorylation for ATP generation and having a very poorly developed mitochondrion. The bloodstream-form trypanosomes are coated with Variant Surface Glycoprotein (VSG), which is anchored to the plasma membrane by glycosyl phosphatidylinositol; a combination of genetic rearrangements and transcriptional switching of the *VSG *expressed enables indefinite evasion of humoral immunity. As the parasitaemia increases, a mechanism resembling quorum sensing [1-3] allows some cells to take on a "stumpy" morphology. Stumpy bloodstream-form trypanosomes are arrested in the G1 phase of the cell cycle, and express some mitochondrial proteins that are not detected in the long slender forms [4].

Differentiation of bloodstream forms into procyclic forms, which multiply in the midgut of the Tsetse fly (reviewed in [5]), can be triggered by various stimuli, including addition of *cis-*aconitate [6], acid treatment, proteolytic stress [7,8] and glucose deprivation [9]. A reduction in temperature stimulates the process but appears not to be essential [10]. A major indicator of procyclic differentiation is the loss of VSG and its replacement by a small family of repetitive proteins called GPEET and EP procyclins. Stumpy forms are pre-adapted for differentiation, and populations replace their surface coat protein synchronously upon subjection to differentiation stimuli. Long slender forms can also differentiate, but do so asynchronously; one possible reason for this could be that differentiation starts in G1 [11]. Procyclic forms obtain their energy mainly by metabolism of amino acids, using several pathways within and outside the mitochondrion, which is much more developed than in bloodstream forms.

Kinetoplastid gene expression is very unusual in that nearly all protein-coding genes are embedded in polycistronic transcription units, individual mRNAs being created by processing [12]. This means that, although global levels of polymerase II initiation may perhaps be reduced upon growth arrest, there is no transcriptional control of the relative amounts of different mRNAs. Instead, regulation of mRNA levels is exclusively post-transcriptional, operating at the levels of mRNA processing and mRNA degradation [13,14]. Final protein levels are further affected by control of translation, and control of protein processing, modification and degradation [15]. The only exceptions to this are the trypanosome VSG and procyclin transcription units, which are still polycistronic, but are transcribed by RNA polymerase I [16]; their transcription is regulated by alterations in chromatin [17] but the mRNAs are also still subject to extensive post-transcriptional control [13,14]. So far, evidence for most mRNAs implicates sequences in the 3'-untranslated regions in control of mRNA decay and translation [13,14]. In a few cases, small sets of co-regulated mRNAs have been shown to contain specific 3'-UTR sequences that are required for regulation, but mostly, searches for such short motifs have been unsuccessful [13,14].

Microarray analyses of the transcriptome of *Leishmania*, comparing the major stages available in culture - amastigotes, and procyclic and metacyclic promastigotes - yielded estimates that 2-3% of genes showed at least 2-fold regulation at the mRNA level [15,18-20]. In a study that analysed expression at 3 time points during the process of differentiation from promastigote to amastigote, 344 regulated protein-coding genes could be grouped into 12 clusters according to the patterns of expression [19].

In previous analyses of the *Trypanosoma brucei *transcriptome, we used arrays of random genomic fragments to compare RNA from cultured bloodstream and procyclic forms, and concluded that approximately 200 of the roughly 8 000 open reading frames in the *T. brucei *genome showed at least 2-fold regulation at the RNA level [21,22]. Another survey, using a targeted oligonucleotide array biased towards genes involved in vesicular trafficking, found that 6% of transcripts were regulated [23]. To find groups of transcripts that are truly co-regulated, however, it is necessary to follow the time course of changes in mRNA abundance. We have now performed a transcriptome analysis of trypanosomes at nine different stages of differentiation, in order to characterise the time course of mRNA changes and to find transcripts that were induced only during differentiation.

## Methods

### Trypanosome culture and RNA preparation

EATRO1125 (clone AnTat 1.1) pleomorphic trypanosomes were cultivated in modified HMI-9 medium [24] with 10% foetal calf serum, at 37°C with 5% CO_2_, with regular dilution so that the density did not exceed 5 × 10^5 ^cells/ml. RNA was isolated from bloodstream trypanosomes in logarithmic growth at 2 × 10^5 ^cells/ml (low density) and at 2 × 10^6 ^cells/ml (high density). To trigger differentiation we made multiple separate flasks containing trypanosomes at 2 × 10^6 ^cells/ml. To each, we added *cis-*aconitate to a final concentration of 6 mM, closed the screw caps, and transferred the flasks to a room at 27°C. The medium had cooled to 30°C after 60 min. Individual flasks were harvested 30 min, 60 min, 12 h and 24 h. At 24 hours, cells in the remaining flasks were centrifuged at 2500 × g and placed in MEM-Pros medium (DTM without *cis-*aconitate and citrate [6]) supplemented with 3% (v/v) hemin and 10% (v/v) heat inactivated FCS. These cultures were harvested 24 h or 48 h later for the 48 h and 72 h time points. To obtain established procyclic forms, the differentiated procyclic trypanosomes were maintained in culture for several weeks, with dilution when the density attained 5 × 10^6 ^cells/ml.

For RNA preparation, trypanosome pellets were extracted using the RNeasy Midi Kit (Qiagen) following the manufacturer's protocol. The quantity of RNA was measured using a Nanodrop ND-1000 3.3, and quality assessed using the Agilent Bioanalyzer.

### DNA and protein analysis

Trypanosomes (2 × 10^6 ^cells) were centrifuged and washed twice in 5 ml PBS. The pellet was resuspended in 200 μl PBS, 2 ml of 70% ethanol:30% PBS was added drop-wise and the cells were stored at 4°C. For use, the parasites were pelleted, resuspended in 1 ml of PBS supplemented with 20 μg RNase A and 50 μg propidium iodide, incubated at 37°C for 30 minutes then analysed by FACSSCAN. Protein electrophoresis, Western blotting and immunofluorescence were done as in [25]; blot b was made without sample heating [26]. Antibodies were to Antat1.1 VSG (from Prof M. Engstler, Darmstadt), tubulin (from Prof K Gull, Oxford), EP repeat (Cedar Lane), PAD1 (from Prof K. Matthews, Edinburgh), aldolase [27] and the exosome component RRP6 [25].

### Microarray hybridisation and image acquisition

We used version 3 of the *Trypanosoma brucei *microarray from the Pathogen Functional Genomics Resource Center - J. Craig Venter Institute. This contains 8594 70 mer oligonucleotides, each representing an open reading frame from the *T. brucei *927 genome, spotted in duplicate onto aminosilane-coated slides. Sample labelling and detection were as previously described [28]. Each condition was hybridized six times (three times per biological replicate) and Cy3 and Cy5 dyes were swapped to minimize dye-bias [29].

For each hybridisation, 10 μg of total RNA were randomly primed with 0.5 μg of random hexamers (Invitrogen Life Technologies), incubated at 70°C for 10 min and placed on ice for 5 min. First strand cDNA was then synthesized using 400 units of Superscript III reverse transcriptase (Invitrogen Life Technologies) in a master mix containing 8.5 μl of 5× Superscript III First-Strand Buffer (250 mM Tris-HCl - pH 8.3, 375 mM KCl, 15 mM MgCl_2_), 40 units of RNaseOUT recombinant ribonuclease inhibitor (Invitrogen Life Technologies) and final concentrations of 1.75 mM DTT, 1.5 mM of d(A, T, G) mix, 0.1 mM of dCTP and 1 mM of Cy3 or Cy5 fluorophore-labelled dCTP (Amersham Biosciences). Samples were incubated at 52.5°C for one hour. 200 units of Superscript III reverse transcriptase were again added and samples were incubated at 52.5°C overnight. Two units of Ribonuclease H (Invitrogen Life Technologies) were added and samples were incubated at 37°C for 20 min. Labelled cDNA was purified using the QIAquick PCR purification kit (QIAGEN), ethanol-precipitated and resuspend in 60 μl double distilled H_2_O. The concentration and dye incorporation were measured in a fluorimeter (Nanodrop ND-1000 3.3).

Slides were pre-hybridized in 5× SSC, 0.1% SDS and 1% BSA at 42°C for 45 min. Meanwhile, the Cy3- and Cy5- labelled cDNAs were mixed and vacuum dried to ~10 μl. The cDNAs were then added to 40 μl of hybridisation buffer (5× SSC, 0.1% SDS, 40% formamide, 0.6 mg/ml salmon sperm DNA), denatured at 95°C for 5 min, placed on ice for 2 min and applied to the pre-hybridised slides. A "lifterslip" (Implen) was affixed and slides were incubated at 42°C overnight in the Slidebooster SB800 hybridisation station (Advalytix). Slides were washed for 10 min each at 50°C in low stringency buffer (2× SSC; 0.5% SDS), at room temperature in medium stringency buffer (0.2× SSC; 0.5% SDS) and in high stringency buffer (0.1× SSC), then N_2 _dried. Microarrays were scanned with ScanArray 5000 (Packard BioScience, Dreieich, Germany) and analyses of resulting images were performed using GenePix Pro 6 software (Axon Instruments, Union City, USA).

### Data pre-processing and clustering

Image acquisition and data analysis were performed as previously described [22,21] using the MCHiPS software package for data normalisation and analysis [30,31]. After subtracting the local background from each single spot, signal intensities were normalised by loglinear regression. All hybridizations showed correlation coefficients higher than 0.71 between the two channels, almost all higher than 0.8 (scatter plots in Additional file 1, Figure S1).

log_2 _transformed data were exported to SAM [32] for multiclass testing (600 permutations). We selected genes that satisfied two conditions. First, the normalised intensity level had to be ≥ 1817.25 in at least one of the conditions. (Maximal signal intensities were of the order of 40000 to 60000; background signals from oligonucleotides representing a few selected unlikely open reading frames on the "wrong" strand were 100-200). Second, the adjusted p-value had to be ≤ 0.01 for at least one of the conditions under study. 1113 oligonucleotides survived this filtering and data from these were used for further analysis. The condition medians of log_2 _transformed ratios for each condition were exported to MeV [33] where genes were hierarchically clustered for overall data visualisation. To obtain individual clusters of genes sharing similar expression profiles across all conditions, data were K-means clustered (Pearson correlation, 50 maximum). The 60 generated clusters were exported to Excel. After retrieving Entrez Gene IDs using the Batch Entrez Tool [34] we used DAVID (Database for Annotation, Visualisation, and Integrated Discovery) [35] to assign Gene Ontology, Interpro, Pfam, Kegg and Pubmed annotations, if available, to the clustered genes. The automatic annotation was then manually checked, with additional data from the literature, including the glycosomal [36,37] and flagellar [38] proteomes, as well as published metabolic pathway information [39,40]. The results are shown in detail in Additional files 2, 3 and 4. (Tables S1, S2 and S3).

### Data validation by Quantitative RT-PCR

For validation of the microarray gene expression data we chose 21 genes that were significantly differentially regulated in one or more conditions covering all possible different expression patterns. Gene-specific primers for each validation candidate were designed, using RNAit [41], to amplify a fragment of 105 +/- 5 bp in each open reading frame. Most of the fragments included the oligonucleotide present on the array; the exceptions were Tb927.8.7680 and Tb09.160.4480. The values for three non-regulated genes, Tb927.3.930, Tb927.8.7680 and Tb927.7.1830, were used as references for the relative quantification. The reaction mixes were checked after amplification, and primer pairs that gave sufficient primer dimers to prevent quantitation were discarded.

EXPRESS SYBR GreenER qPCR SuperMixes were used with the Two-Step qRT-PCR kit (Invitrogen Life Technologies). Following the manufacturer's instructions, we diluted 1 μg RNA to 7 μl with nuclease-free water (Ambion), added 1 μl of 10× DNase reaction buffer with MgCl_2 _(Thermo Fisher Scientific Inc), then incubated with 1 μl DNase I, RNase-free (1 u/μl) (Thermo Fisher Scientific Inc) at 37°C for 30 min. 25 mM EDTA (Thermo Fisher Scientific Inc) was then added before DNase I inactivation at 65°C for 10 minutes. The entire sample was then mixed with 4 μl of 5× Vilo reaction mix, 2 μl of 10× Superscript Enzyme mix (Invitrogen Life Technologies) and 4 μl of nuclease-free water (Ambion). After gentle mixing, the samples were incubated at 25°C for ten minutes followed by 42°C for 60 minutes. The reaction was terminated at 85°C for 5 minutes and samples were diluted 1:10. For each qRT-PCR reaction, 5 μl of EXPRESS SYBR GreenER qPCR SuperMix Universal (Invitrogen Life Technologies), 0.4 μl of 10 μM each of forward and reverse primers, 2.6 μl of nuclease-free water (Ambion) and 2 μl of diluted cDNA were pre-mixed and transferred into a LightCycler 480 Multiwell Plate 384 (Roche Diagnostics). Plates were sealed with LightCycler 480 sealing foil (Roche Diagnostics) and pre-incubated at 95°C for 5 min (ramp rate of 4.8°C/s) for denaturation on the LightCycler 480 (Roche Diagnostics). PCR was done for 40 cycles of 95°C 10 seconds (ramp rate of 4.8°C/s), 55°C 20 sec (ramp rate of 2.5°C/s), 72°C 20 sec (ramp rate of 4.8°C/s), with a signal read at the end of each cycle. A final melting curve to check fidelity was done from 95°C 5 sec (ramp rate of 2°C/s), 65°C 1 min (ramp rate of 2°C/s) with 5-10 signal acquisitions every 1°C up to 97°C.

## Results

### Characteristics of transforming trypanosomes

In this work, we analysed differentiation of the EATRO 1125/LUMP 581 strain of *Trypanosoma brucei brucei*, which was first isolated in 1966 from a bushbuck (*Tragelaphus scriptus*) in Uganda [42]. Our trypanosomes were obtained from the laboratory of Prof. P. Overath (Tübingen) in 1990, and were of serotype AnTat1.1 [42]. The cells were stored in liquid nitrogen since 1990 and, when cultured, were maintained at densities below 5 × 10^5^/ml.

Stumpy-form parasites can be obtained either from mice or using soft agar plates. However, in order to perform array hybridisations without any amplification step we needed samples of at least 3 × 10^8 ^trypanosomes, and to avoid alterations in RNA during harvesting we needed to be able to obtain pure trypanosomes in a single centrifugation step. We therefore sought a procedure by which we could obtain reproducible differentiation in liquid culture. It was previously reported that pleomorphic EATRO1125 growing in liquid suspension culture differentiate into stumpy forms, arresting at the G1 phase of the cell cycle as they enter stationary phase [1]. The cells we used grew exponentially (division time of 6 h or less) at densities below 7 × 10^5^/ml, with long slender trypomastigote morphology. If they were then left in the same medium, growth slowed so that the density the next day was about 2 × 10^6^/ml. We made various attempts to obtain pure stumpy forms *in vitro*, by prolonging the period of high density culture. After a further day, the maximum density attained was 2.5 × 10^6^/ml; these cultures were a mixture of shorter, more rounded cells (resembling stumpy forms) and variable proportions of dead and dying cells. Although these cultures contained viable cells capable of rapid differentiation (data not shown), the presence of dying cells made them unsuitable for routine use.

To obtain RNA for microarray hybridisation, we used the following procedure. A fresh stock of EATRO1125 was thawed and grown at low density until sufficient parasites were available for the experiment. Long slender bloodstream forms were harvested at a density of 2 × 10^5^/ml (low density, logarithmic growth). For differentiation, trypanosomes were allowed to grow to 2 × 10^6^/ml (high density), then immediately treated with 6 mM *cis*-aconitate and allowed to cool to 27°C. Samples were taken 30 min, 60 min, 12 h and 24 h after this, to detect immediate and more gradual changes. At 24 h the cells were centrifuged, resuspended (at 27°C) in MEM-Pros medium, which contains proline as the major energy source. Samples were taken again at 48 h and 72 h. A culture that had been maintained for several weeks after transformation was used as a source of established procyclic trypanosomes.

**Figure 1:**
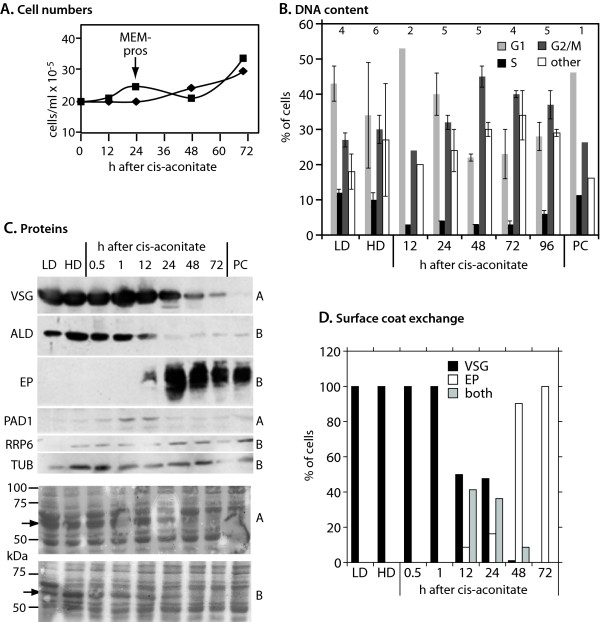
**Growth and differentiation characteristics of theEATRO1125 trypanosomes used in this study**. (A) Growth of the two cultures used for RNA preparation. (B) DNA content during differentiation, measured by FACSScan. The mean and standard deviation are shown, with the number of replicates above the bars. G1 cells had diploid DNA content, G2/M cells 4×, and the S phase intermediate amounts. Cells in the "other" category mostly had more DNA, with peaks at 6× and 8×; a few (under 20% of this category) had less than 2×. (C) Western blots showing expression of various proteins during differentiation. Two blots, A and B, were used; a portion of each, stained for total protein using Ponceau red, is shown at the bottom of the Figure, with molecular weight markers indicated. The arrow points to a thick bloodstream-specific band that migrates at the expected position of VSG. The blot used in each case is indicated on the right. The upper panels show immunoreactivity with: VSG: variant surface glycoprotein Antat1.1; ALD: aldolase; EP: EP procyclin; PAD1: stumpy-specific transporter; RRP6: component of the exosome; TUB: tubulin. (D) Differentiating trypanosomes were stained for VSG and EP procyclin. The percentages of cells with VSG, EP procyclin and both are shown. For the 12 h, 24 h, 48 h and 72 h points, 100 cells were counted. The other samples showed uniform staining over many fields.

The experiment was done twice for RNA preparation: growth curves for these two cultures are illustrated in Figure 1A. After transfer to differentiation conditions, little or no growth occurred for 48 h, after which trypanosome growth resumed. Assays of DNA content in similar cultures showed that the high-density bloodstream forms were not arrested in G1: instead, there were fewer G1 cells than in the low-density cultures, and an increase in abnormal forms (Figure 1B) with 6N and 8N DNA content (not shown). G1 cells were more prominent at 12 h. At 12, 24 and 48 h, the proportion of S phase cells was significantly lower than in growing bloodstream forms. An increase in G2/M cells was however evident at 48 h and 72 h consistent with the start in cell division. Analyses of the numbers of nuclei and kinetoplasts in the cultures led to similar conclusions (not shown). The presence of relatively large numbers of 6N and 8N cells in the differentiating population indicates that some cells were unable to divide properly after transfer to the new conditions. From these results, it is clear that in our *in vitro *cultures, the initiation of division was much slower than has been reported for stumpy cells, which start dividing 12 h after *cis-*aconitate addition [43].

During differentiation, EP procyclin was detectable 12 h after addition of *cis*-aconitate. VSG and aldolase had decreased after 24 h (Figure 1C); immunofluoresence analysis showed that, as expected [6,11], the exchange of surface coats was not synchronous (Figure 1D). The stumpy-form marker PAD1 [26] was up-regulated 1-12 h after *cis-*aconitate treatment, then declined (Figure 1C). This, together with the DNA analysis, confirms that the high density trypanosomes were mostly not stumpy forms, and suggests that a stumpy-like population accumulated in response to *cis-*aconitate addition. In procyclic cells, the distance between the nucleus and kinetoplast is shorter than in bloodstream forms. In our cells this distance showed a detectable decrease at 12 h, and reached the final procyclic level at 48 h (not shown). During stumpy-form differentiation, kinetoplast movement was detectable at 6 h and complete at 18 h [44].

The average yield of total RNA was 39 μg/10^8 ^cells, (0.4 pg/cell), with no reproducible variation between treated groups. This value is similar to that reported previously for bloodstream forms [45]. We expected to obtain more RNA from established procyclics than from bloodstream forms, but did not: this could be connected to the different cell densities used. Our high-density bloodstream forms did not have the low RNA levels reported previously for stumpy forms [46].

### Over 1000 RNAs show significant changes in abundance during trypanosome differentiation

**Figure 2:**
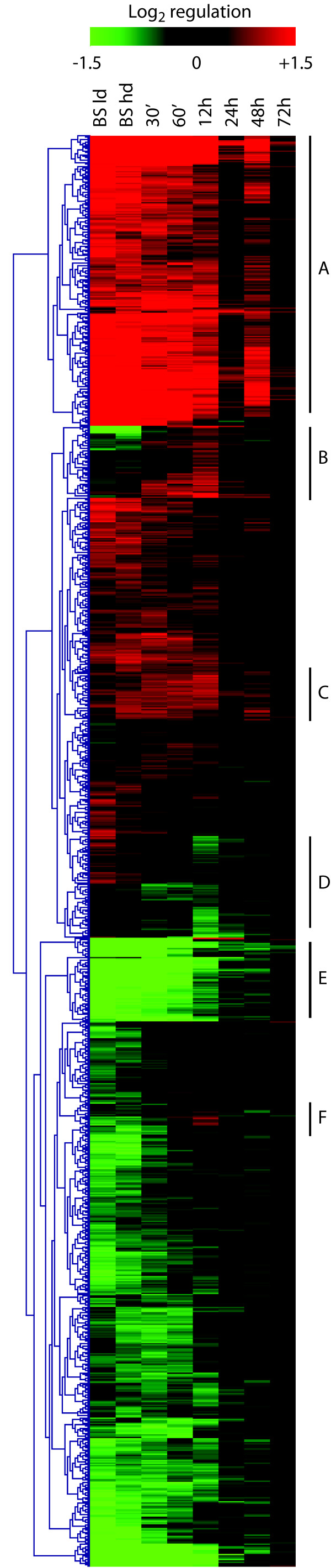
**Colour-coded display of the 1113 regulated genes identified in the analysis**. Regulation factors were Log_2 _transformed and genes were hierarchically clustered according to distances between different conditions, using MeV [33]. The coding is shown above the graph: red indicates higher expression than mature procyclics, green lower. The scale is from ± 2.8-fold, so all values above this give saturated colour and moderate regulation is visible (threshold about 1.6-fold). A few selected profiles are indicated by letters on the right (see text).

The RNAs from two independent biological replicates of the whole transformation procedure were hybridised to oligonucleotide arrays, using a two-colour analysis with established procyclic trypanosomes as the reference. We selected spots that had intensities significantly above background in at least one condition, with an adjusted p-value equal to, or less than 0.01. We did not apply a threshold for the degree of regulation required. Using these criteria, 1113 regulated genes were identified. To obtain an overview of regulation patterns, the regulation factors were Log_2 _transformed and ratios for each condition were sent to MeV [33] where genes were hierarchically clustered. This method takes into account the distances from point to point, rather than the overall profile. The results are shown in Figure 2. It was evident that in addition to the expected genes showing specific up-regulation in either bloodstream-forms (e.g. A) or procyclic forms (e.g. E), there were also groups of genes showing either up-regulation (B, C, F) or down-regulation (D) during differentiation. It was also clear that the change in medium at 24 h caused a transient, partial reversion in gene expression towards a bloodstream-form pattern; this could be a side-effect of stress. Regulation of several genes was checked by quantitative reverse-transcription-PCR. In all cases where the results were technically acceptable, they agreed with those from the array. In general, it appeared that the microarray under-estimated the extent of regulation (Additional file 5, Figure S2).

To obtain individual clusters of genes sharing similar expression profiles, data were K-means clustered based on Pearson correlated distance. Using this method, genes with a similar overall pattern of expression (but not necessarily quantitatively similar levels of regulation) are grouped. The reason to do this was that we were particularly interested in seeing which genes responded at particular stages of differentiation. Most of the clusters were similar to those obtained previously, except for some genes showing relatively low regulation. Some of these clusters are described in detail below, and the whole set is available in Additional files 2, 3 and 4 (Tables S1, S2 and S3).

The number of regulated mRNAs that we found is considerably higher than previously reported. This can partially be attributed to improvements in technology, since the methodology has become more sensitive, with a larger dynamic range than observed previously: the amplitude of regulation detected by microarray in some cases approached that seen by Northern blotting or quantitative RT-PCR. Another reason for the apparent increase in regulated genes is the use of p-values without any threshold concerning the extent of regulation; previous analyses have always set a regulation threshold, usually of 2-fold or more. Most importantly, however, the large numbers of samples used increased the statistical power of the analysis, and we were able to see transient alterations during differentiation.

### Annotation of regulated RNAs

We analysed the functions of all regulated genes by domain searching, and checked annotations manually with reference to the literature (see Methods and Additional files 2, 3 and 4. With some notable exceptions, most clusters contained genes with a variety of functions. In the discussion below, we pick out some notable examples of co-regulated genes with similar functions, and, for known differentiation markers, compare our results with previous reports. In our discussion, mRNA levels will be regarded as equivalent to procyclic if they are between 70% and 130% of the procyclic level (± log_2 _0.5). Our analysis has the following limitations: (A) There is only one oligonucleotide per open reading frame in the array; since the oligos have different melting temperatures we could not use the fluorescence intensities to obtain information concerning the relative abundances of different mRNAs. (B) The oligos were designed using the 927-strain genome sequence; since this does not include the minichromosomes or telomeres some of the oligonucleotides may hybridise with RNAs other than the one for which they were designed. (C) We used a different trypanosome strain from 927, so sequence variations may prevent oligonucleotide hybridisation. (D) Most functional designations for genes are only tentative. In our discussion the word "putative" is omitted in the interests of readability. (E) In the discussion we will assume - unless specifically stated - that increases, or decreases in mRNAs result in corresponding changes in the levels of the encoded proteins. This is an over-simplification, since control of translation and of protein degradation might either accentuate or counteract changes in mRNA abundance. Also, small changes, even at the protein level, may have no biological consequences.

### Regulation of mRNAs encoding surface proteins

**Figure 3:**
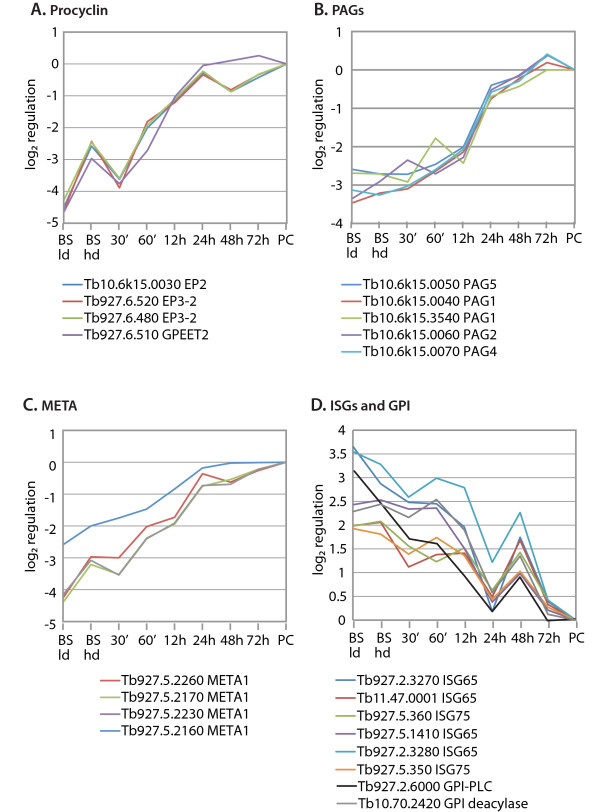
**Regulation of mRNAs encoding surface proteins**. The log2 regulation ratio (sample/procyclic) is plotted on the Y axis for the different time points given on the X axis. Time points are not to scale. The data points are not shown but are joined together by straight lines; each array spot is represented by a different colour as indicated in the legend below, which indicates the GeneDB locus number and gene identity. Genes are grouped according to function, as indicated above the graphs; individual genes within one graph may be assigned to different regulatory clusters. (A) Procyclins, the procyclic-form-specific surface coat; (B) Procyclin-associated genes (PAGs), co-transcribed with the *EP *and/or *GPEET *genes; (C) Genes homologous to *Leishmania META *genes; (D) Bloodstream-form specific invariant surface glycoprotein genes (*ISG*) and two genes involved in bloodstream-form specific GPI anchor metabolism.

The mRNAs encoding GPEET and EP procyclins are known to increase dramatically upon differentiation, as a consequence of induction of transcription (by RNA polymerase I) and stabilisation of the mRNA; GPEET appears earlier than EP [47,48]. The 927 genome has an *EP *gene cluster on chromosome 10, containing *EP1 *and *EP2 *genes, with downstream *PAGs1*, *5, 2 *and *4*, and clusters on chromosome 6 with *GPEET*, *EP3 *and *PAG3 *genes. All of these apart from the *EP1 *and *PAG3 *genes are represented on the array. The co-regulation of all the procyclin genes was striking (Figure 3A); the approximately 25-fold increase measured here approaches that previously reported by cDNA cloning and Northern blotting ([49] and references therein). These mRNAs already increased in the high-density bloodstream forms. Although we have not found any previous report of this precise phenomenon, *EP *mRNA has been seen in stumpy-form mRNA [50] and it also increases upon Aphidicolin-induced cell-cycle arrest of bloodstream forms [51], and after other treatments that block nuclear DNA synthesis or modify DNA [52]. Evidence so far indicates that the effect is specific to polymerase I-transcribed protein-coding genes [52].

Upon addition of *cis-*aconitate, *EP and GPEET *mRNAs briefly fell then started to rise rapidly, attaining 50% of established procyclic levels within 12 h. This time-course is very similar to that seen during differentiation of pure stumpy-form trypanosome populations [50,43] except that the brief decrease at 30 min was not previously reported.

The *PAG1, 2, 4 *and *5 *genes are co-transcribed with *EP2 *so transcriptional regulation alone is expected to result in an increase during differentiation. Indeed, although regulation was slightly less dramatic than that of the procyclins, the *PAG *mRNAs induction kinetics were very similar to those of *EP2 *(Figure 3B). The fact that *PAG *mRNAs did not increase at high density contradicts the previous suggestion that this effect is caused by changes in polymerase I transcription, suggesting instead that it may be post-transcriptional. An array spot representing an isolated *PAG *gene downstream of *CRAM*, at a transcriptional convergence point, was also co-regulated, but the significance of this is unclear: this oligonucleotide might hybridise with conventional *PAG *mRNA from EATRO1125.

Three oligonucleotides specific to *META *genes were co-regulated with the procyclin mRNAs while a fourth showed slightly different regulation (Figure 3C). The *META *RNA is up-regulated in metacyclic *Leishmania*; over-expression increases *Leishmania *virulence [53]. Its role in trypanosomes has not been investigated but the expression pattern found here suggests that it might be associated with procyclin expression or trafficking.

While the procyclins increase, the VSG decreases, but the Antat1.1 VSG is not represented on the array. Another set of bloodstream-form-specific plasma membrane proteins, the ISGs, is however shared by all antigenic variants and in different isolates. These mRNAs responded extremely rapidly to *cis-*aconitate, were at procyclic levels within 24 h, rebounded after the medium change, and had decreased again by 72 h (Figure 3D). Both VSG and the procyclins are anchored in the plasma membrane by a glycosyl phosphatidylinositol (GPI) anchor. The mRNA encoding GPI-specific phospholipase C, which has a role in VSG shedding, decreased with similar kinetics to the Tb927.2.3270 *ISG65 *mRNA, while that encoding the GPI deacylase, which is responsible for a bloodstream-form-specific GPI anchor modification, followed the Tb927.5.1410 *ISG65 *mRNA. The result for *GPIPLC *mRNA is compatible with that previously reported for differentiation from stumpy forms [43].

It has previously been reported that mRNAs encoding some components of the vesicular transport system are more abundant in bloodstream forms [23]; we found that many relevant mRNAs were about 2-fold more abundant in bloodstream forms, reducing to procyclic levels between 1 h and 24 h after differentiation started (Additional file 6, Figure S3).

### Changes in energy metabolism

Bloodstream trypanosomes metabolise glucose mainly to pyruvate (90%) and glycerol (10%), using a glycolytic pathway localised predominantly in a microbody, the glycosome. Procyclic trypanosomes, in contrast, have several alternative pathways for energy generation [39,40]. Glucose is metabolised in the glycosome to 1,3 bisphosphoglycerate, which is converted to phosphoenol pyruvate in the cytosol. The phosphoenol pyruvate can then take several routes, including cytosolic conversion to lactate or to alanine; glycosomal conversion to succinate; or conversion to acetate in the mitochondrion [54]. The procyclic medium that we used has proline as a major energy source: this is converted to succinate.

**Figure 4:**
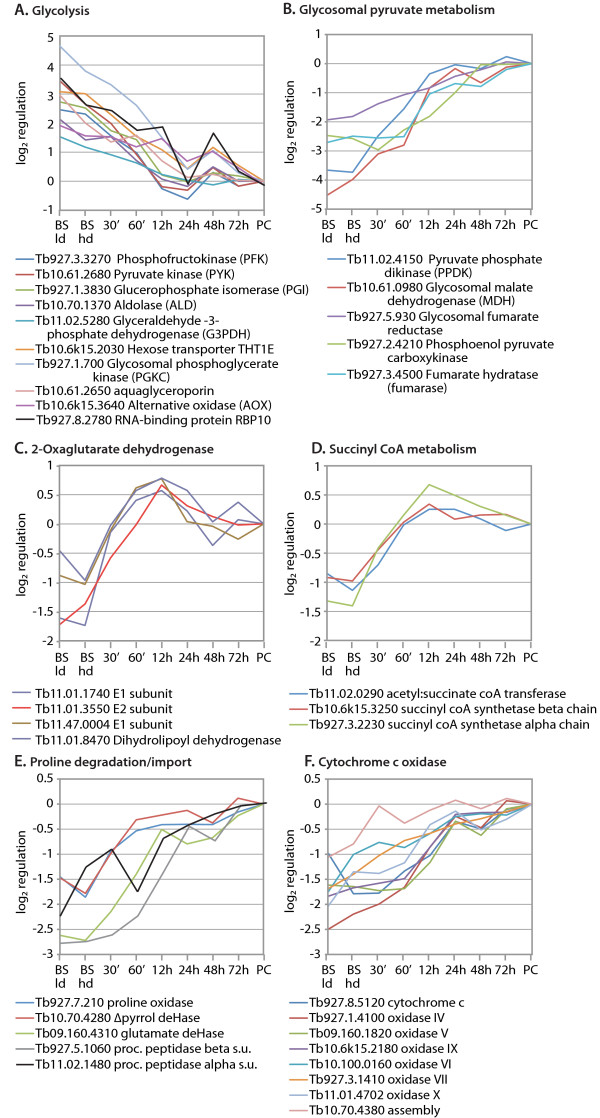
**Regulation of mRNAs involved in energy metabolism**. Plots were made as in Figure 3. (A) Genes involved in glycolysis and glycerol metabolism, up-regulated in bloodstream forms; (B) mRNAs involved in pyruvate conversion to succinate in the glycosome; (C) the mitochondrial oxaglutarate dehydrogenase complex (dihydrolipoyl dehydrogenase is also a component of the pyruvate dehydrogenase complex); (D) enzymes involved in succinyl coA metabolism; (E) proline degradation within the mitochondrion; (F) Cytochrome c and cytochrome c oxidase; the 10.70.4380 protein is a homologue of yeast Cox11p, which delivers copper to the COX1 subunit.

The mRNAs encoding many of the glycosomal enzymes are known to be more abundant in bloodstream forms than in procyclics, and there is less *PYK *mRNA in short stumpy forms than in long slender trypomastigotes [1]. The regulated mRNAs encoding enzymes required for glucose and glycerol metabolism decreased only slightly in our high density culture, but addition of *cis-*aconitate caused most of them to fall two-fold within 60 min and several had reached procyclic levels by 12 h. (Figure 4A.) Other mRNAs encoding proteins involved in bloodstream-form energy metabolism, including those encoding a hexose transporter, the mitochondrial alternative oxidase, and an aquaglyceroporin [55], took longer to decrease, but in no case was the medium switch necessary for regulation (Figure 4A). The mRNA encoding RBP10, an RNA-binding protein which may control the abundance of several mRNAs required for glycolysis (M. Wurst and R. Queiroz, ZMBH, unpublished results), varied in parallel with its possible targets (Figure 4A). The mRNAs for glycosomal pyruvate metabolism increased as those for glycolysis decreased (Figure 4B). The array spot for cytosolic *PGKB *mRNA - which is known to increase during differentiation - showed no change. This oligonucleotide has the potential to cross-hybridise with the *PGKC *mRNA, which shows opposite regulation to *PGKB*. We suspect therefore that the apparent lack of *PGKB *regulation in the array is a consequence of cross-hybridisation by a large excess of *PGKC *mRNA in the bloodstream-form probe.

The mRNAs encoding components of the 2-oxaglutarate dehydrogenase complex were changed roughly 4-fold during differentiation, with striking co-regulation: the amounts had increased to procyclic levels within 30 min of *cis-*aconitate treatment (Figure 4C). Transcripts for succinyl CoA metabolism showed a similar rapid response to *cis-*aconitate (Figure 4D). This suggests that one of the first metabolic responses to *cis-*aconitate is a switch to allow generation of ATP via the succinyl CoA pathway, fed either via 2-oxaglutarate or more directly from pyruvate. It is notable that the mRNAs for enzymes for proline degradation (Figure 4E) were induced before, not after, the switch to high-proline medium: clearly they are not induced by the presence of substrate.

All nuclear-derived mRNAs encoding cytochrome oxidase subunits are much more stable in procyclic trypanosomes than in long slender bloodstream forms [56]. These mRNAs were strongly up-regulated within 24 h (Figure 4F and Additional file 7, Figure S4A and S4B), consistent with the rapid up-regulation previously reported [50]. Coordinate up-regulation of threonine dehydrogenase with NADH-dependent fumarate reductase and a subunit of the F1 ATPase was more gradual (Additional file 7, Figure S4C). The only annotated gene for mitochondrial protein import that showed significant regulation encodes the beta subunit of the signal peptidase [57]. This mRNA increased more slowly than those encoding many mitochondrial enzymes (Figure 4E). (The alpha subunit data included here were reproducible, but just below our intensity-level cut-off.) Levels of proteins encoded by kinetoplast DNA may be limited by the activity of the editing complex; in our analysis the RNA-binding protein RBP16 and a mitochondrial DEAD-box helicase showed developmental regulation (Additional file 7, Figure S4A and S4B).

### Lipid metabolism and sterols

The glycosyl phosphatidylinositol anchor of VSG differs from that of the procyclins in lacking acylation of the inositol ring. The four-fold decrease in the mRNA encoding the deacylase occurred rather late, starting only after 12 h (not shown). Although bloodstream trypanosomes are capable of lipid biosynthesis, procyclic trypanosomes have higher activity [58,59] and RNAs encoding several enzymes of lipid biosynthesis increased correspondingly (not shown).

*T. brucei *bloodstream forms can obtain cholesterol from the serum, whereas procyclic trypanosomes synthesise ergosterol. Of the regulated mRNAs of this pathway, four were approaching procyclic levels within 60 min, but hydroxymethylglutaryl-CoA reductase and lanosterol 14-alpha-demethylase [60] mRNAs attained this level only after 24 h (Additional file 8, Figure S5). The former enzyme is generally considered to be rate-limiting for sterol biosynthesis.

### Growth-correlated adjustments

**Figure 5:**
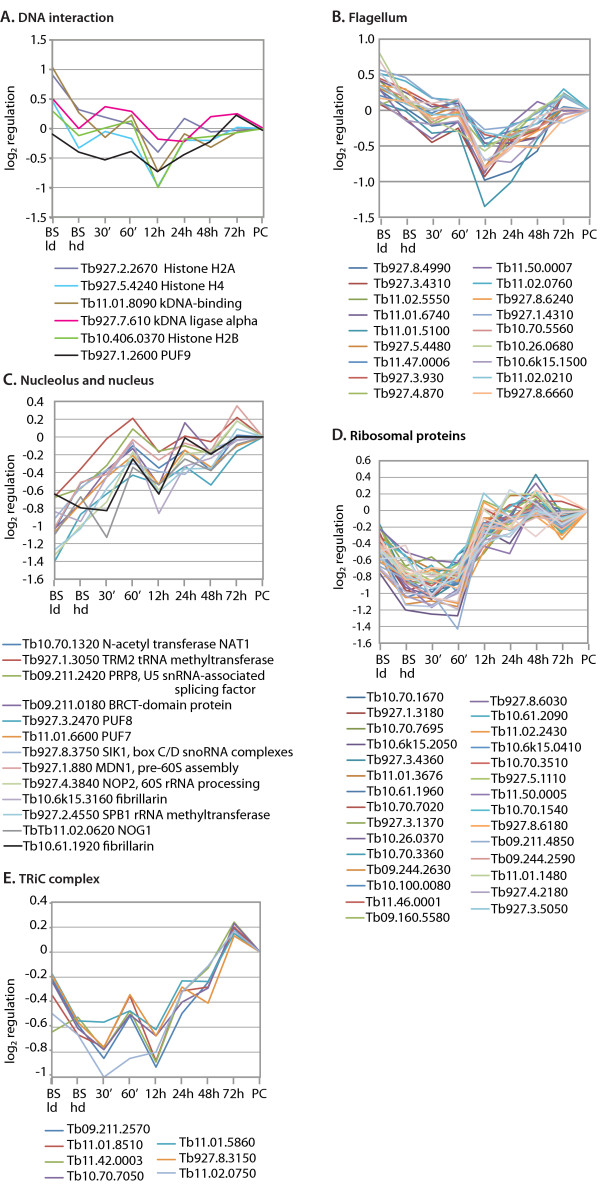
**Coordinate regulation of mRNAs implicated in various aspects of cell division and macromolecular biosynthesis, plotted as in Figure 3**. For annotation of the ribosomal and flagellar proteins see Additional file 3, Table S2.

Stumpy-form trypanosomes are arrested in G1 and histone gene expression correlates with growth [50]. In our experiments the mRNAs encoding histones H2A, H2B and one variant of histone 4 were 25-50% decreased in the high-density trypanosomes, and reduced further at 12 h (Figure 5A). Coordinate down-regulation was also seen for two mRNAs involved in kDNA replication: one of them, kDNA ligase alpha, is maximal in G1/S in *T. brucei *[61]. Dividing trypanosomes also have to synthesise a new flagellum: many mRNAs required for this were specifically reduced at 12 h (Figure 5B). All of this is consistent with the observed low level of S-phase cells at this time point.

Cells that are not growing are expected to have a relatively low requirement for components of macromolecular synthesis; on the other hand, protein synthesis is required during differentiation, since new proteins are needed. It was indeed previously reported that overall protein synthesis pauses in the first 24 h after differentiation [62]. Figure 5C shows the transcript patterns for 11 nucleolar proteins affecting rRNA processing and modification; although differences were overall less than 2-fold, the coordinated patterns suggest the differences are meaningful. The most dramatic cluster of the whole dataset, however, is shown in Figure 5D: these are 29 ribosomal protein mRNAs that were decreased at high density, and had risen again by 12 h. Other mRNAs in this cluster included those encoding translation factor eIF5a and a subunit of nascent polypeptide-associated complex. The increase in ribosomal protein RNAs was followed by an increase in mRNAs encoding most components of TriC (Figure 5E), a hetero-oligomeric complex that helps to fold newly-synthesised proteins [63]. We selected these and a few other clusters, downloaded predicted untranslated regions [64], and looked for common elements using Trawler [65], without much success.

### Transcripts that rise or fall specifically during the differentiation process

**Figure 6:**
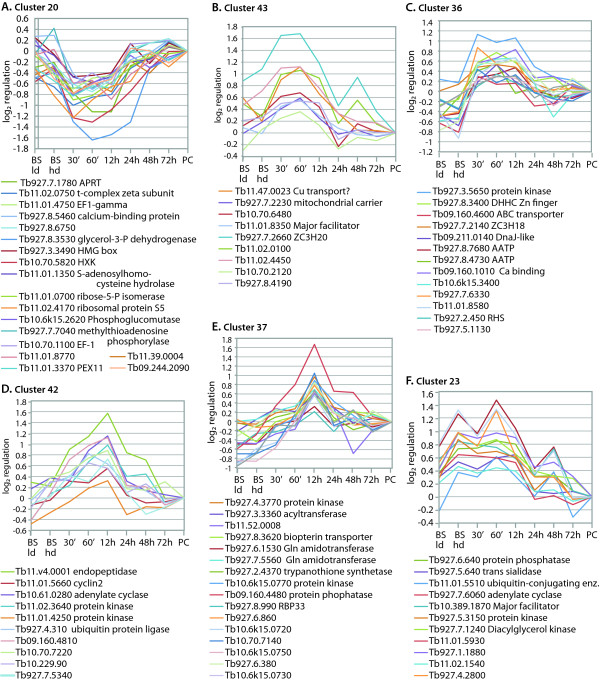
**Six regulatory clusters showing specific changes during differentiation, plotted as in Figure 3**. (A) APRT - adenine ribophosphoryl transferase, HXK: hexokinase. (C) AATP: amino-acid transporter, RHS: retrotransposon hotspot.

Many of the regulated mRNAs described so far have known functions, and/or had previously been shown to show preferential expression in either bloodstream or procyclic forms. A major aim of this study was to identify mRNAs that were affected only during the differentiation process. Several different clusters had this pattern; six are illustrated in Figure 6. Most of the annotated genes in cluster 20 (Figure 6A), with reduced expression at 30 min -12 h, are expected to be needed for growth: perhaps the other genes in the cluster are too. The clusters in Figure 6B-F, in contrast, include mRNAs that are up-regulated during differentiation. The mRNAs that are rapidly up-regulated (Figure 6B, C) are particularly interesting since they may encode proteins that are required for early stages of differentiation: likely regulators include RNA-binding proteins of the CCCH zinc finger family, two protein kinases, a protein phosphatase and a ubiquitin conjugating enzyme. Two other clusters shown (Figure 6D, E) include four potential protein kinases, another ubiquitin ligase, a cyclin, an RNA-binding protein and a protein phosphatase. Finally, Cluster 23 includes mRNAs that are increased in the high-density culture and are already decreasing by 12 h.

### In vitro differentiation is delayed relative to differentiation from stumpy forms

The accompanying paper by Kabani *et al *[44] describes a transcriptome analysis for EATRO1125 trypanosomes that were allowed to develop into stumpy forms in mice before transfer to procyclic conditions. The parasites from blood were purified before RNA was made, then allowed to recover briefly in *in vitro *culture. The hybridisations were competitive (with a procyclic control) in our case, while the data for the *in vivo *model were obtained by hybridisation of single probes: this technical difference precludes quantitative comparisons. Finally, our data were filtered based on p-value alone whereas the Kabani *et al *data were filtered using a threshold for differential regulation in addition to p-value [44].

**Figure 7:**
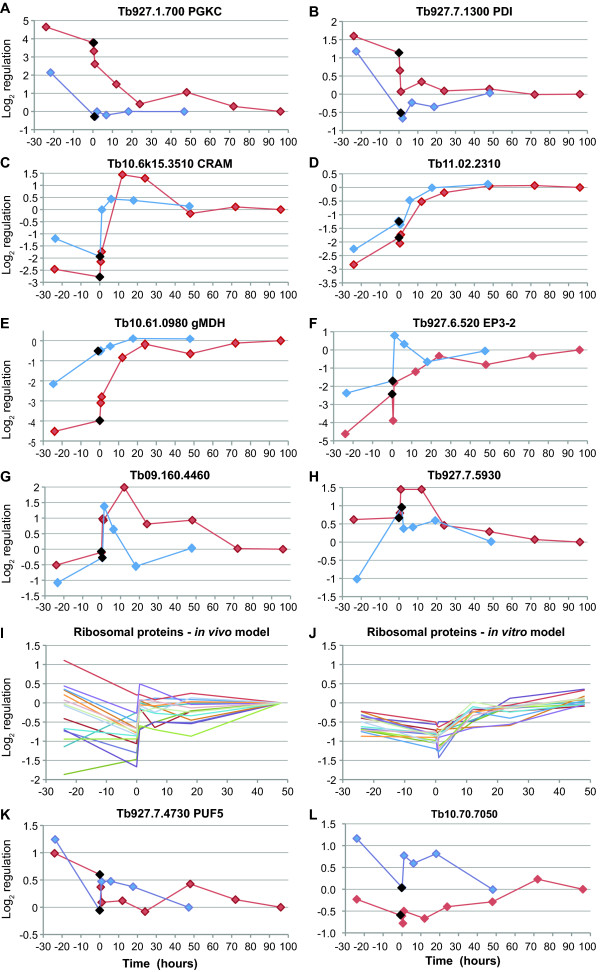
**Comparison of data for selected genes during differentiation, starting from either high density culture, or stumpy forms **[44]. To enable the comparison, results from Kabani *et al *[44] were converted so that all mRNA levels were expressed relative to the amounts 48 h after the initiation of differentiation, so assuming that the 48 h values were equivalent to established procyclic forms. Values for the low-density cultures and long slender bloodstream forms are shown arbitrarily at time = -24 h. For our results, established procyclic values are shown arbitrarily at 96 h. Times are shown to scale. With the exception of I and J, results from Kabani *et al *[44] are in blue and those from this paper, in red; the diamonds for stumpy forms or high-density bloodstream cultures are black. In panels (I) and (J), results for 15 different ribosomal protein genes are shown, for differentiation from stumpy forms (I) or from dense cultures (J).

A preliminary comparison of both datasets revealed that of 355 spots that were judged to be up-regulated (relative to procyclics) in the *in vivo *analysis, only 76 were identified in our purely *in vitro *experiments. Similarly, only a minority of the genes identified as regulated *in vitro *were present in the *in vivo *dataset. A comparison of the results for selected genes is shown in Figure 7. In most cases, the trends were similar, although the amplitude of regulation was higher in the *in vitro *data. The discrepancies between the datasets could be due to genuine differences between bloodstream trypanosomes grown in mice, and in culture, or could be consequent to the technical issues listed above.

One difference between the dataset that is almost certainly biologically meaningful is that changes in the *in vitro *differentiation system were delayed by up to 24 h relative to those seen during differentiation from stumpy forms. *PGKC *(Figure 7A) was already at procyclic levels in stumpy forms, but attained that level only after 24 h in our experiments. Other examples, showing varying delays, are shown in Figure 7 B-F. Three transporter-like proteins, PAD1, PAD6 and PAD8 are implicated in *cis-*aconitate transport [26], and PAD1 is up-regulated at both protein and mRNA levels in stumpy forms. In our experiments, the *PAD1 *mRNA and protein were increased 60 min and 12 h after addition of *cis-*aconitate, (Cluster 26, see Figure 1C and Additional file 4, Table S3). This supports the notion that at least some of the *in vitro *parasites become "stumpy-like" *after *addition of *cis-*aconitate. *PAD6 *and *PAD8 *are also enriched in stumpy forms (see accompanying paper); in our experiments, however, *PAD8 *showed little regulation while PAD6 was specifically reduced in the high-density and 30 min samples (Clusters 1 and 34 respectively, see Additional file 4, Table S3). Figure 7G and 7H show results for two mRNAs that we identified as being regulated during differentiation, but which were already elevated in stumpy trypanosomes. A comparison of ribosomal protein gene regulation showed that as expected, the expression was generally suppressed in stumpy forms, but increased as soon as *cis-*aconitate was added and the cells re-entered the cell cycle (compare Figure 7I with 7J); Finally, for some genes (e.g. Figure 7K, L) very little correlation between the two datasets was discernable.

## Discussion

### Are stumpy forms made during in vitro differentiation?

The results described here document the coordinated regulation of many functionally related sets of mRNAs during trypanosome differentiation. Many of the changes in metabolic pathway mRNAs were already well known: the novelty here lay only in seeing the precise timing of the changes. Other information was however entirely new. In particular, the pattern of regulation of the growth-related mRNAs suggested that after the differentiation stimulus, the trypanosomes - which were already not growing - further reduced resources for cell division. After initial synthesis of mRNAs required to adjust energy metabolism and surface coat composition, an increase in rRNA processing preceded synthesis of additional ribosomal proteins, facilitating synthesis of other new proteins from the changed transcriptome. It has previously been claimed that switches in trypanosome gene expression do not depend on growth arrest or transition through the G1 phase - in other words, formation of stumpy forms is optional (see e.g. [66]). This claim was, however, based on light microscope morphology and expression of surface proteins alone. (Also, as in our experiments, outgrowth of a small sub-population of arrested cells could not be ruled out.) Our transcriptome results suggest that, on the contrary, the dense *in vitro *cultures underwent a transition to stumpy-like gene expression over the first 12 hours of exposure to *cis-*aconitate. Although the initial increase in procyclin mRNA upon *cis-*aconitate addition was far too rapid to depend on cell cycle progression, the full switch to a growing procyclic-form transcriptome was completed only after the population had stopped growth and expressed some stumpy-form-specific mRNAs.

### Post-transcriptional regulons

For several years, it has been evident that in animal cells and yeast, post-transcriptional control is superimposed upon regulation at the level of RNA polymerase: mRNAs that need to be synthesised in order to activate a particular pathway must also be stabilised or degraded in a coordinated fashion. In the simplest case, for a particular pathway, under a particular physiological condition, the mRNAs have similar half-lives, determined by shared sequence elements, which are usually located in the 3'-untranslated regions and bound by specific protein (or RNA) factors. This is the concept of "post-transcriptional regulons" [67,68]. A global analysis of mRNA decay rates in *Saccharomyces cerevisiae *revealed that although there was no simple relationship between mRNA stability and abundance [69], mRNAs encoding different components of a wide variety of protein complexes had similar half-lives [69]. These included the ribosome and various enzyme complexes, such as succinate dehydrogenase and 2-oxoglutarate dehydrogenase, which also showed coordinate regulation in trypanosomes. Coordinated regulation of mRNA stability for genes involved in ribosome biogenesis was also seen in another yeast study [70]. Human cells too show differing decay rates for different protein functional categories [71]. In *Plasmodium*, mRNA half-lives vary globally during intra-erythrocytic growth, but the patterns of variation are similar for genes in broad functional groups [72]. Analyses of mRNAs that are targeted by specific RNA binding proteins have similarly shown that often, the proteins encoded by the target mRNAs are functionally related [67,68,73].

Trypanosomes are exceptional in that microarray results concerning steady-state mRNA levels give direct insights into post-transcriptional regulation and likely differences in mRNA half-lives. We expect that if regulatory sequence elements are bound by specific proteins, these may themselves show changes in expression. Indeed, several of the clusters include mRNAs encoding RNA-binding proteins (e.g. Figures 4A, 6B, C and 6E, and 7K), and in one case (RBP10, Figure 4A) we have preliminary evidence that the protein indeed targets the co-regulated mRNAs (M. Wurst and R. Queiroz, ZMBH, unpublished results). The functional groups that we have identified show particularly tight correlation in regulation patterns and should provide an excellent starting point in the search for regulatory factors.

## Conclusion

Our results suggest that trypanosome genes form post-transcriptional regulons in which mRNAs with functions in particular pathways, or encoding components of protein complexes, show almost identical patterns of regulation. The differentiation of *in vitro *cultivated bloodstream trypanosomes is delayed by about 12 hours relative to the differentiation of stumpy forms taken from mice.

## Authors' contributions

R.Q. did the practical work and most of the analysis described in this paper, under the supervision of J.H. and C.C C.B. supervised the trypanosome culture. K.F. contributed to the data analysis. C.C. did the manual annotation and functional grouping of genes, and wrote most of the manuscript with R.Q. All authors read and approved the final manuscript.

## Supplementary Material

Additional file 1 Figure S1 Click here for file

Additional file 2 Table S1 Click here for file

Additional file 3 Functional Groups Click here for file

Additional file 4 Clusters Click here for file

Additional file 5 RT-PCR Click here for file

Additional file 6 Vesicular transport Click here for file

Additional file 7 Mitochondrian Click here for file

Additional file 8 Sterols and thiols Click here for file
