# Supplementary material for: Transcriptome analysis of differentiating trypanosomes reveals the existence of multiple post-transcriptional regulons
Source: BMC Genomics. 2009 Oct 26;10:495. doi: 10.1186/1471-2164-10-495 (PMC2772864; doi:10.1186/1471-2164-10-495)
Supplement: Additional file 1 — Figure S1 [file 1471-2164-10-495-s1.pdf]

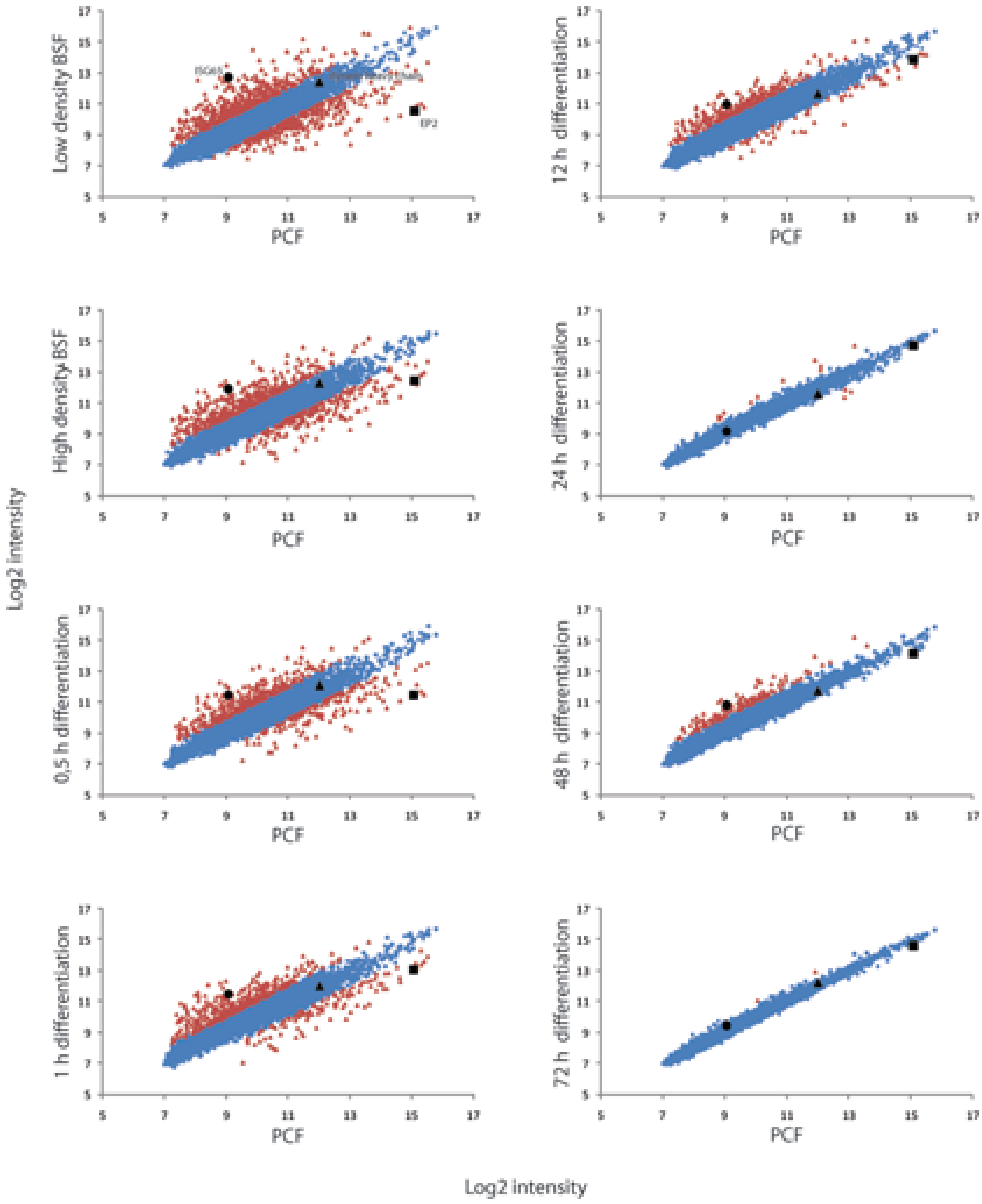

Figure S1
Scatter plots of significant transcriptome changes during differentiation. In each plot, X-axis represents log2 values of the median of fitted intensities of the specific condition and the Y-axis, the log2 values of the normalised intensities of the control (PCF total RNA). Each spot represents a single ORF. Significantly regulated ORFs (absolute log2 expression value ≥ 1) are shown in red. Genes not significantly regulated are shown in blue. The black triangle shows the intensities of the ORF coding for dynein heavy chain (Tb927.3.930) during differentiation. This gene was used for relative quantification of the qPCR data since there were no significant signal intensity differences between condition and control during differentiation. Expression values of an ISG65 gene (Tb927.2.3270) and EP2 procyclin gene (Tb10.6k15.0030) are represented by the black circle and square, respectively.
