# Supplementary material for: Transcriptome analysis of differentiating trypanosomes reveals the existence of multiple post-transcriptional regulons
Source: BMC Genomics. 2009 Oct 26;10:495. doi: 10.1186/1471-2164-10-495 (PMC2772864; doi:10.1186/1471-2164-10-495)
Supplement: Additional file 5 — RT-PCR [file 1471-2164-10-495-s5.pdf]

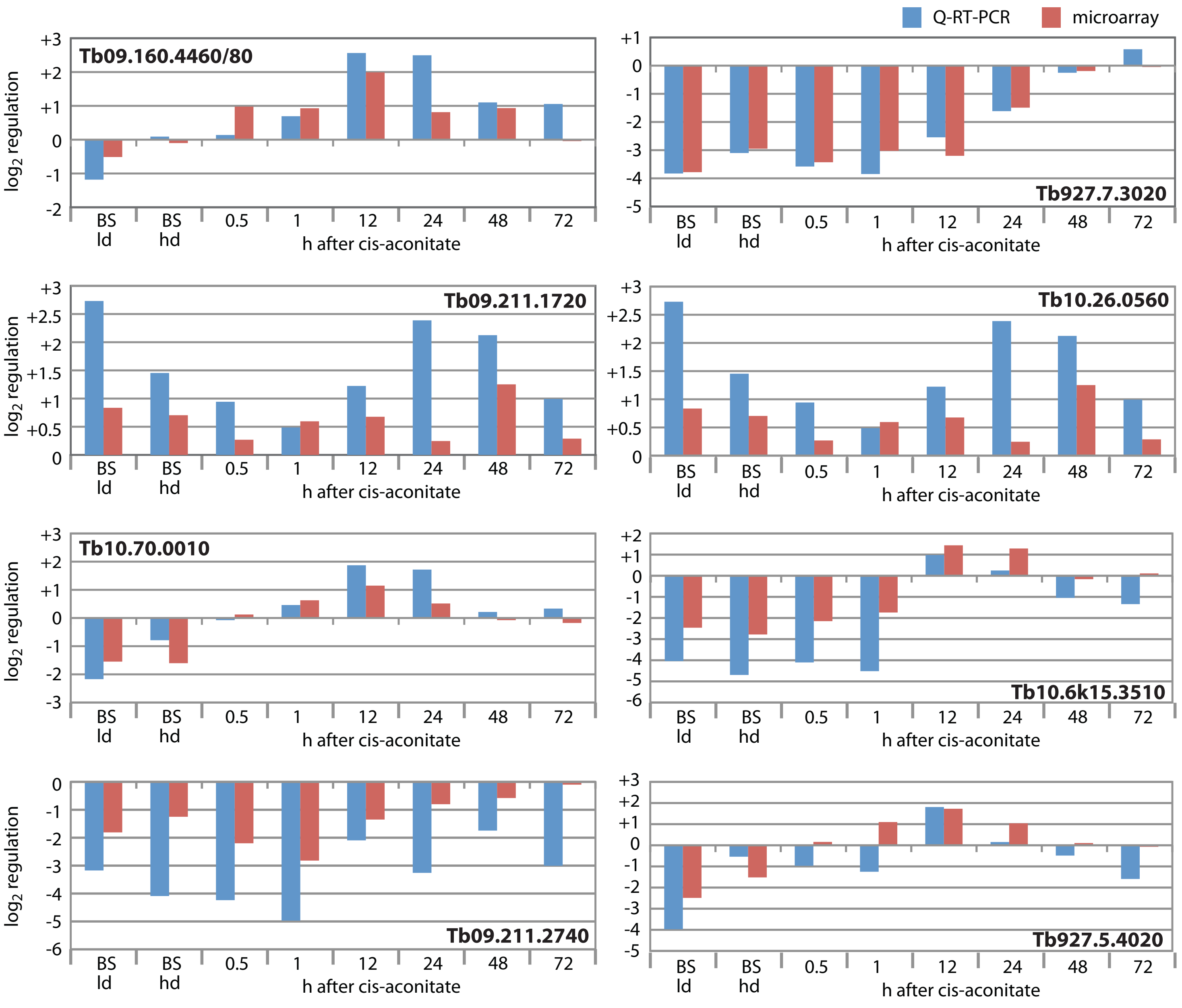

Figure S2

Comparison of gene expression values from microarray and quantitative rtPCR results. Log2 of normalised gene expression values from qrtPCR relative quantification and from microarray results are plotted on the charts. Gene-specific primers were designed for 21 significantly differentially regulated genes in one or more conditions using RNAit (Redmond, 2003) , to amplify a fragment of 100+/- 25 bp in each open reading frame (Tb09.160.4460/80, GAAAACCCACACTGGTGCTT / ATGTGGACGCAG-GAAGAAAC; Tb927.7.3020, TCAGCCTTCAATTCCCAATC / GGGTTTCACTGTCGCCTTTA; Tb09.211.1720, TTCTACTCCTTGGGACGAGTG / ATGTCTGCAAAGGGAAGACG; Tb10.26.0560, CAGCGGTGTTCCTATTCGTC / GCTTGAAGATTTCAGGGGTG; Tb10.70.0010, ATAGCTGT-GGCGTCGCTTT / GCGCCTCCAATATTGATGAT; Tb10.6K15.3510, TCATATGCGACAACGAGGTT / TTACGGGTACGGCAAAAGAT; Tb09.211.2740, TGTACATGCTGTCAAAAATGAGG / CCATAAGGGCACGTTTTCC; Tb927.5.4020, ACTTCATATTCGCACCGACC / GCGGAAGTTTACGCAGTTGT). Most of the fragments included the oligonucleotide present on the array; the exceptions were Tb09.160.4460/80 and Tb10.6k15.3510. The value for a non-regulated gene, Tb927.3.930, was used as reference for the relative quantification.
