# Supplementary material for: Transcriptome analysis of differentiating trypanosomes reveals the existence of multiple post-transcriptional regulons
Source: BMC Genomics. 2009 Oct 26;10:495. doi: 10.1186/1471-2164-10-495 (PMC2772864; doi:10.1186/1471-2164-10-495)
Supplement: Additional file 6 — Vesicular transport [file 1471-2164-10-495-s6.pdf]

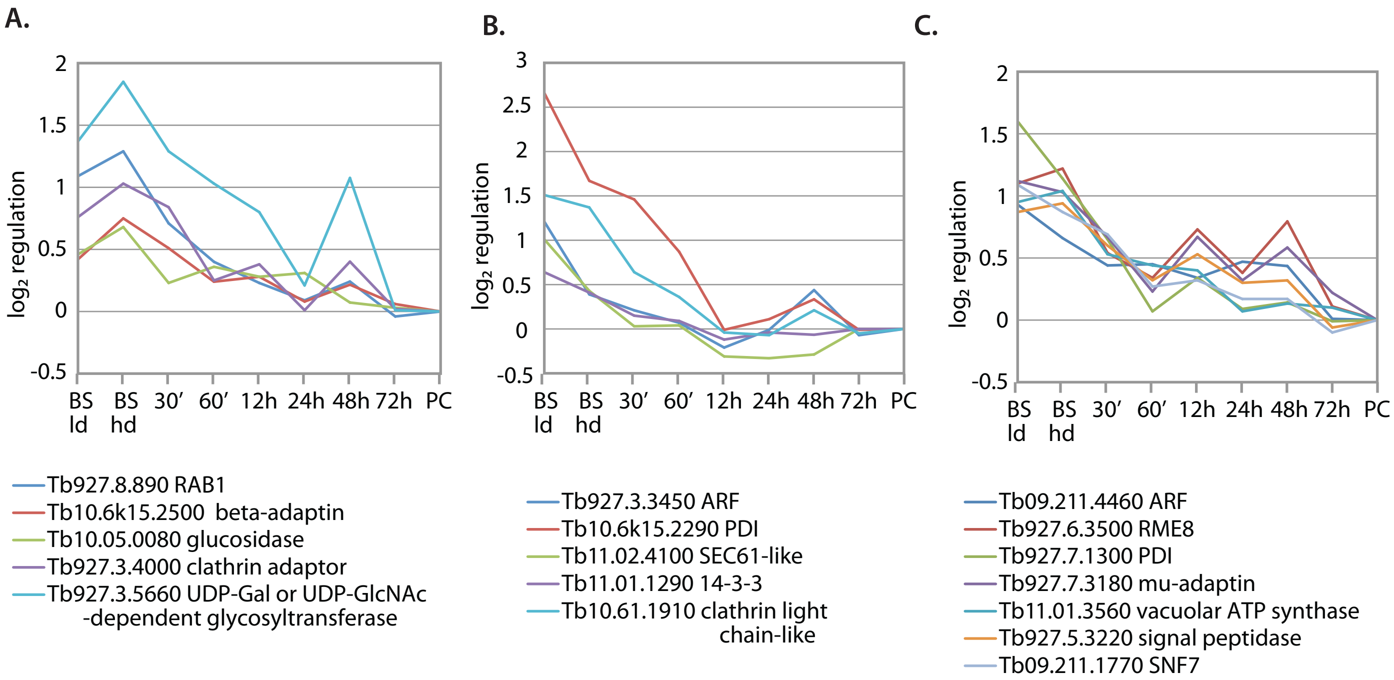

Supplementary Figure S3: Regulation of mRNAs invovled in vesicular transport. A, B, and C show three different patterns.
