# Supplementary material for: Transcriptome analysis of differentiating trypanosomes reveals the existence of multiple post-transcriptional regulons
Source: BMC Genomics. 2009 Oct 26;10:495. doi: 10.1186/1471-2164-10-495 (PMC2772864; doi:10.1186/1471-2164-10-495)
Supplement: Additional file 8 — Sterols and thiols [file 1471-2164-10-495-s8.pdf]

A. Steroid metabolism

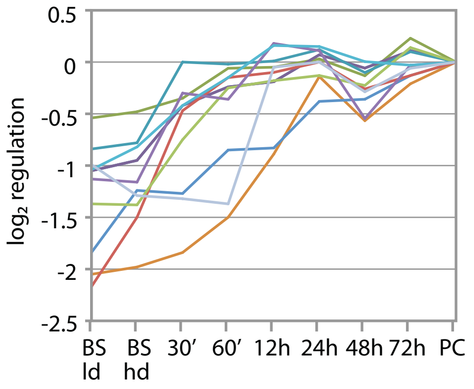

- Tb927.7.3360 farnesyl pyrophosphate synthase
- Tb10.61.2745 mevalonate diphosphate decarboxylase
- Tb927.4.4070 mevalonate kinase
- Tb11.02.4080 lanosterol 14-alpha-demethylase
- Tb927.6.4540 3-hydroxy-3-methylglutaryl-CoA reductase
- Tb927.3.4650 C-8 sterol isomerase
- Tb10.6k15.3820 sterol 24-C-methyltransferase
- Tb927.8.7120 farnesyltransferase, squalene synthase
- Tb11.02.0780 squalene monooxygenase
- Tb927.8.6110 hydroxymethylglutaryl-CoA synthase

B. Redox, polyamine, trypanothione

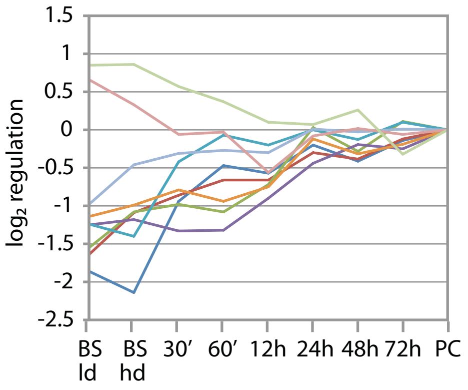

- Tb11.02.5400 cystathionine beta-synthase
- Tb10.389.1360 gamma-glutamylcysteine synthetase
- Tb09.v1.0380 spermidine synthase
- Tb09.160.4280 tryparedoxin peroxidase
- Tb09.211.3330 cystathione gamma lyase
- Tb927.6.4470 S-adenosylmethionine decarboxylase regulator?
- Tb10.70.7770 similar to glutathione S-transferase
- Tb11.01.7550 iron superoxide dismutase
- Tb927.7.112 glutathione peroxidase-type tryparedoxin peroxidase

Supplementary figure S5. Regulation of mRNAs for (A) sterol and (B) redox metabolism.
